# Molecular and functional characterization of detrusor PDGFRα positive cells in spinal cord injury-induced detrusor overactivity

**DOI:** 10.1038/s41598-021-95781-2

**Published:** 2021-08-11

**Authors:** Ken Lee, Sang O Park, Pil-Cho Choi, Seung-Bum Ryoo, Haeyeong Lee, Lauren E. Peri, Tong Zhou, Robert D. Corrigan, Andrew C. Yanez, Suk B. Moon, Brian A. Perrino, Kenton M. Sanders, Sang Don Koh

**Affiliations:** 1 Department of Physiology and Cell Biology, University of Nevada School of Medicine, Reno, NV 89557 USA; 2 Department of Emergency Medicine, Konkuk University School of Medicine, Seoul, South Korea; 3 Department of Emergency Medicine, Kangbuk Samsung Hospital, Sungkyunkwan University, Seoul, South Korea; 4 Department of Surgery, Seoul National University Hospital, College of Medicine, Seoul National University, Seoul, South Korea; 5 Department of Surgery, School of Medicine, Kangwon National University, Chuncheon, South Korea

**Keywords:** Cell biology, Physiology

## Abstract

Volume accommodation occurs via a novel mechanism involving interstitial cells in detrusor muscles. The interstitial cells in the bladder are PDGFRα^+^, and they restrain the excitability of smooth muscle at low levels and prevents the development of transient contractions (TCs). A common clinical manifestation of spinal cord injury (SCI)-induced bladder dysfunction is detrusor overactivity (DO). Although a myogenic origin of DO after SCI has been suggested, a mechanism for development of SCI-induced DO has not been determined. In this study we hypothesized that SCI-induced DO is related to loss of function in the regulatory mechanism provided by PDGFRα^+^ cells. Our results showed that transcriptional expression of *Pdgfra* and *Kcnn3* was decreased after SCI. Proteins encoded by these genes also decreased after SCI, and a reduction in PDGFRα^+^ cell density was also documented. Loss of PDGFRα^+^ cells was due to apoptosis. TCs in ex vivo bladders during filling increased dramatically after SCI, and this was related to the loss of regulation provided by SK channels, as we observed decreased sensitivity to apamin. These findings show that damage to the mechanism restraining muscle contraction during bladder filling that is provided by PDGFRα^+^ cells is causative in the development of DO after SCI.

## Introduction

As the bladder fills with urine the volume increases, but during much of the filling period, intravesical pressure remains low^1^. This accommodation occurs even though stretch-dependent ion channels in smooth muscle cells (SMCs) would tend to make contraction of the detrusor a probable response to stretch^2,3^. However, during bladder filling, non-voiding contractions (NVCs) which are transient increases in intraluminal pressure, occur in cystometric records from all species including human. NVCs appear to correspond to localized contractions that are also observed in ex vivo bladder preparations and have been termed ‘spontaneous phasic contractions’, ‘micromotions’ or ‘transient contractions’^4–8^. Transient contractions (TCs) increase as bladder filling proceeds^4^. Prior experiments have shown that TCs are initiated by stretch-dependent non-selective cation channels expressed by detrusor SMC^2,3^. Inward currents generated by these channels depolarize SMCs and activate L-type Ca^2+^ channels, causing generation of Ca^2+^ action potentials, Ca^2+^ entry into SMCs and contraction. Action potentials propagate to other SMCs within the same muscle bundle, but do not spread to adjacent muscle bundles.

Several studies suggest that TCs initiate afferent nerve activity and provide a major source of the sensory information conveyed to the central nervous system during bladder filling^9–12^. A recent study has clearly demonstrated the link between TCs and sensory output from bladder^4^. Increased TCs may correspond to the sensory and mechanical behaviors associated with detrussor overactivity (DO). Normal bladders have the means to restrain development of TCs, but experiments have not been sufficiently rigorous to reveal the mechanisms responsible for restraining bladder excitability and the development of TCs during filling.

We discovered a novel mechanism involving interstitial cells in detrusor muscles. Interstitial cells of the bladder were previously identified as c-Kit^+^ cells and thought to provide excitatory input to the detrusor^13,14^, but more recent immunohistochemical evaluation showed few c-Kit^+^ cells, other than mast cells, are found in bladders of several species^15^. In fact the major population of interstitial cells in the bladder is platelet-derived growth factor receptor alpha positive cells (PDGFRα^+^ cells). These cells provide inhibitory regulation of detrusor muscles due to activation of small conductance Ca^2+^-activated K^+^ channels (SK channels)^16–20^. Inhibitory regulation is enhanced by purines and by stretch, making it an ideal mechanism for control detrusor excitability and restraining the development of TCs during bladder filling. Rigorous confirmation of the hypothesis that inhibitory regulation during bladder filling is provided by PDGFRα^+^ cells would be to demonstrate pathological conditions where DO develops in association with loss or remodeling of PDGFRα^+^ cells. Therefore, we evaluated the status of PDGFRα^+^ cells in spinal cord injury (SCI) animal models that develop DO.

Clinical manifestations of SCI-induced bladder dysfunction involve a combination of storage and voiding problems. A myogenic origin of DO after SCI has also been suggested due to abnormal muscle reactivity (“the myogenic hypothesis”)^21^ without studying precise mechanisms for DO after SCI. In this study we investigated the mechanisms of DO using murine SCI model.

## Results

**Figure 1:**
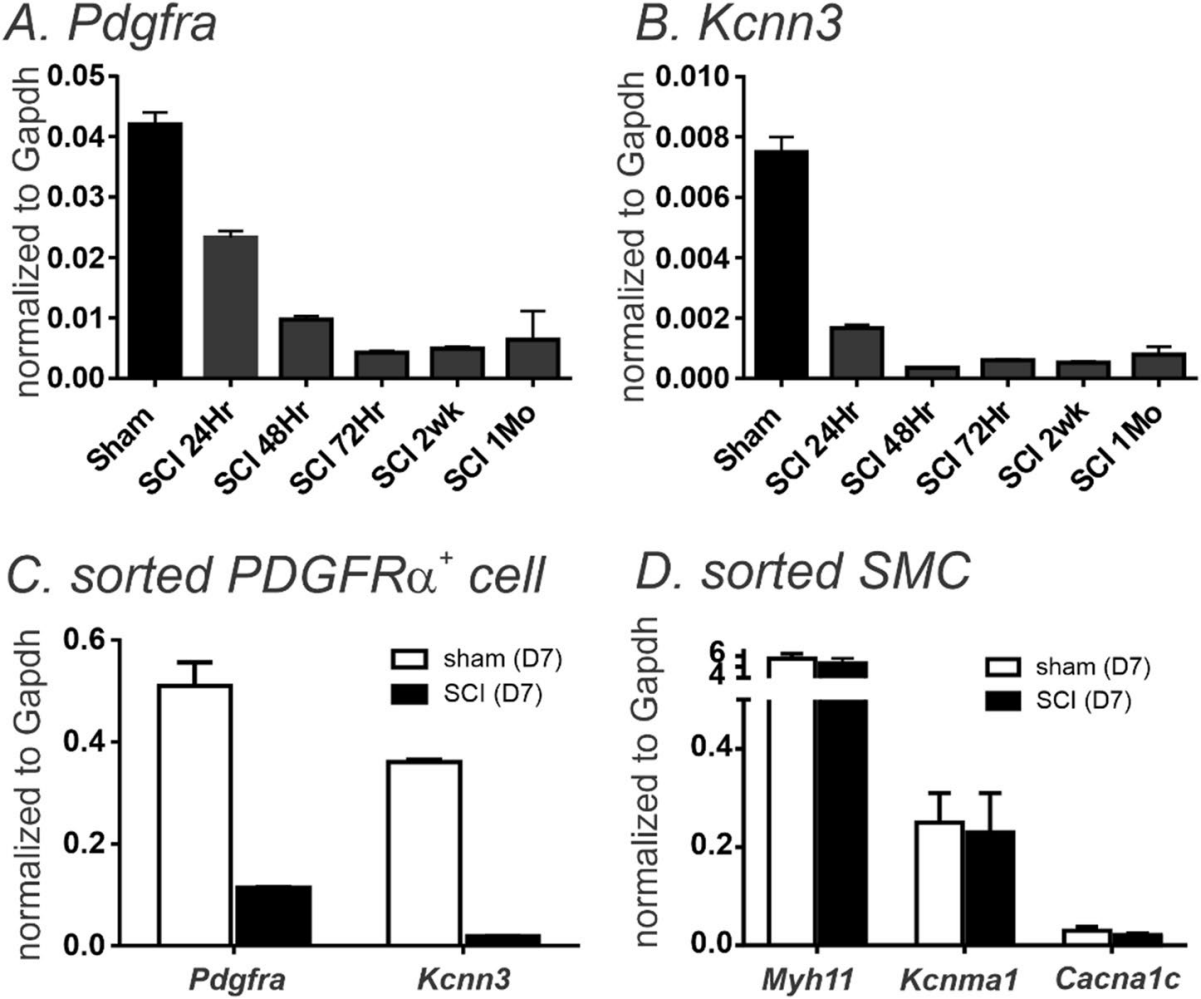
Quantitative analysis of transcripts in control and after SCI. **(A,B) Pdgfra** and *Kcnn3* transcripts are decreased up to 1mo after SCI in detrusor muscles. (**C**) Transcripts of *Pdgfra* and *Kcnn3* from sorted PDGFRα^+^ cells in SCI (D7) decreased compared to control (D7). (**D)** Transcripts including *Myh11, Kcnma1* and *Cacna1C* from sorted SMCs showed no significant change in SCI (D3). Error bars denote standard deviation from n = 4.

**Figure 2:**
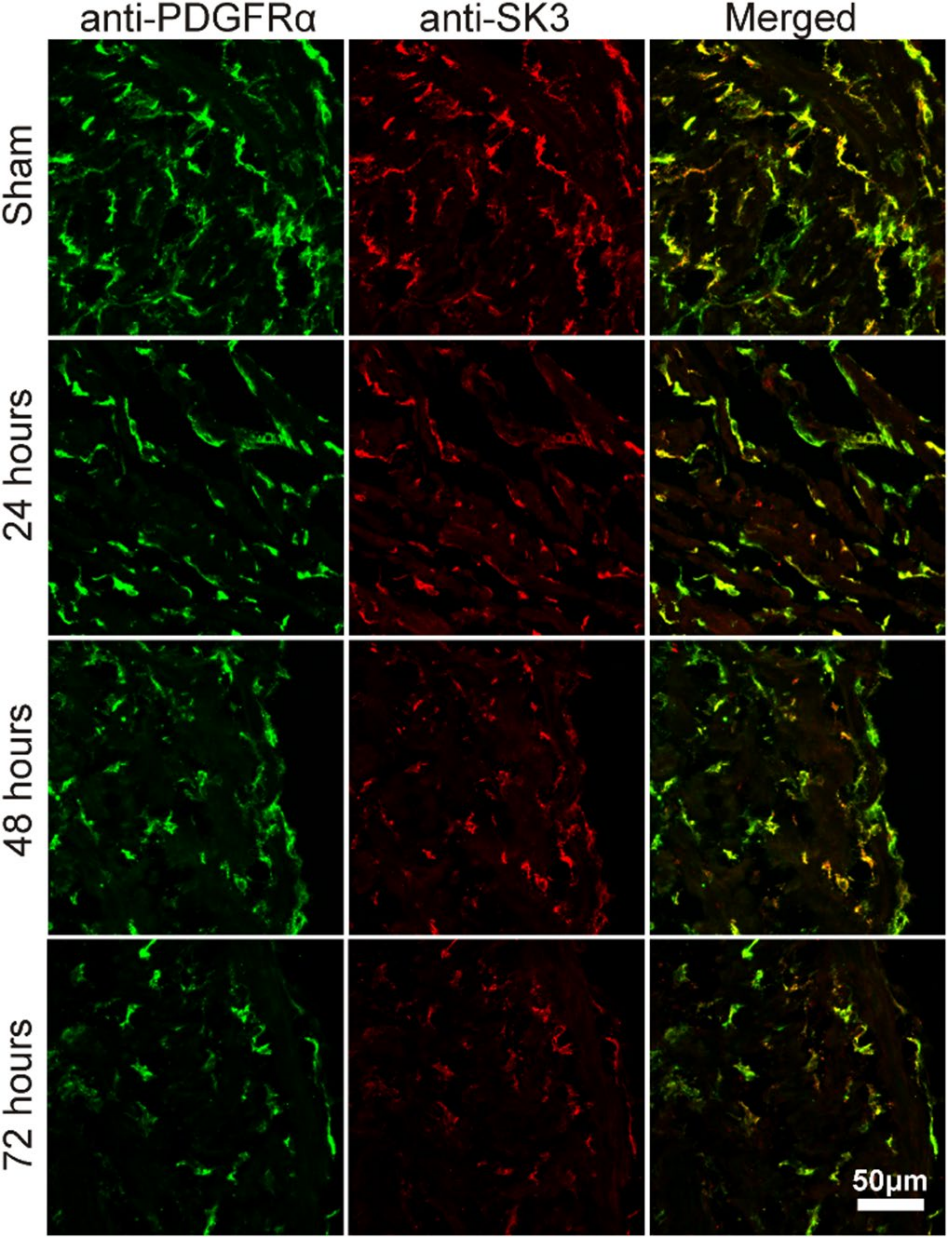
PDGFRα immunoreactivity of detrusor muscle layer in control and SCI. **I**mmunoreactivity of PDGFRα (green) and SK3 (red) in control (sham). SCI (24 h, 48 h and 72 h).

**Figure 3:**
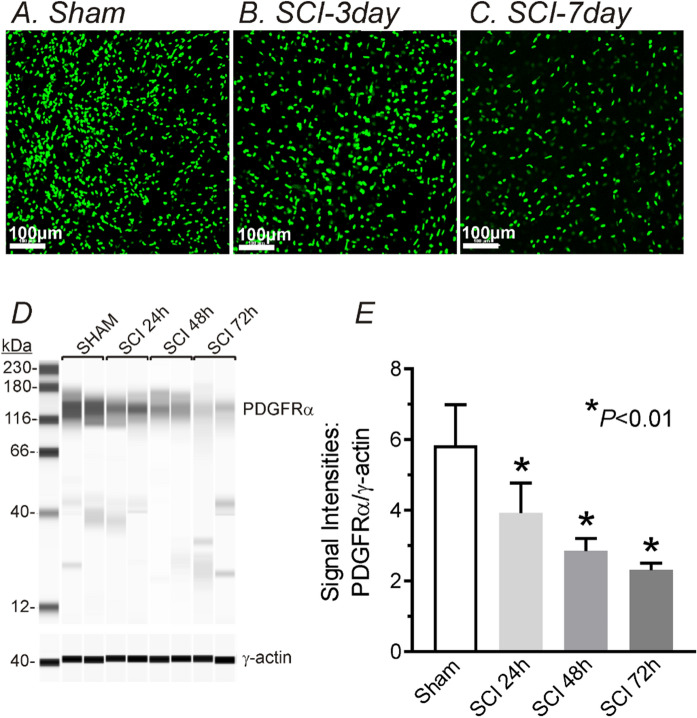
Density of PDFGRα/eGFP cells and microcapillary electrophoresis and immunodetection of PDGFRα by WES in control and SCI detrusor muscles. **(A–C)** Far fewer eGFP^+^ nuclei (reporter for PDGFRα^+^ cells) were found in detrusor muscles after SCI. (**D)** Representative Wes full length gel image of PDGFRα expression in murine detrusor muscles following SCI. 100,000 × *g* pellet, 1 µg/lane, γ-action was used for normalization. (**E)** Normalized signal intensities of PDGFRα by g-actin following SCI periods.

**Figure 4:**
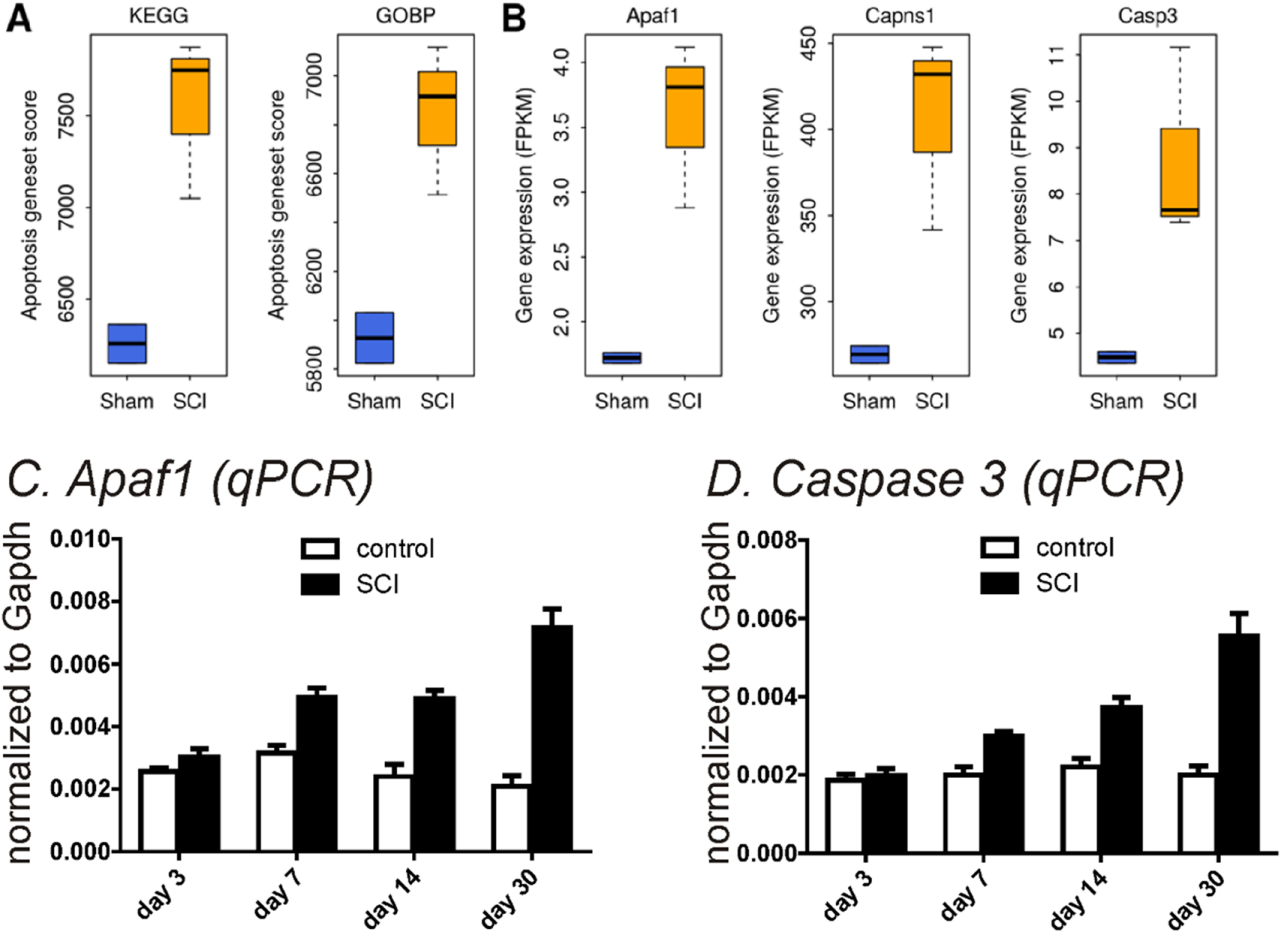
SCI -induced alteration in apoptosis-related genes by RNA-seq **(A,B)** and qPCR **(C,D)**. (**A**) Apoptosis geneset scores upregulated in SCI for both the KEGG and GOBP definitions. (**B)** Expression of three apoptosis-related genes were upregulated in SCI. (C,D)*.* qPCR from sorted PDGFRα^+^ cells in SCI up to 30 days showed an increased in apoptosis-related transcripts (*Apaf1* in (**C)** and *Caspase3* in (**D**)).

1. Changes in transcriptional expression of SCI-induced detrusor muscles
  We harvested detrusor muscles from control (sham) mice and 1, 2, 3, 7, 14 and 30 days (D1-D30) after SCI. *Pdgfra and Kcnn3* expression were decreased in detrusor muscles 24 h after SCI. Reduced *Pdgfrα* and *Kcnn3* expression persisted for at least 1 month after SCI (n = 4, Fig. 1A,B). *Pdgfra* and *Kcnn3* expression also decreased in sorted PDGFRα^+^ cells SCI D7, as compared with sorted PDGFRα^+^ cells from control mice (n = 4, Fig. 1C). We also isolated and sorted smooth muscle cells (SMCs) from smMHC/eGFP mice. Expression of *Myh11* (SM myosin heavy chain), *Kcnma1* (BK α_slo_) and *Cacna1C* (Cav1.2) were unchanged in SMCs after SCI (n = 4, Fig. 1D).
2. Changes in protein expression in SCI-induced detrusor muscles
  Three approaches were used to characterize changes in protein expression. Firstly, immunohistochemistry was used to examine the expression and distribution of PDGFRα and SK3 immunoreacitvity in SCI. PDGFRα and SK3-like immunoreactivity decreased in detrusor muscles after SCI in a time dependent manner (Fig. 2). We also used PDFGRα/eGFP mice and counted the number of nuclei containing eGFP in control and after SCI. Nuclei with eGFP decreased after SCI (D3 and D7) compared to control. Since bladder distension can occur after SCI, nuclei with eGFP per unit area could be misleading. Therefore, we normalized the number of eGFP positive nuclei against the total area of the bladders (e.g. 63 mm^2^ in control vs 111 mm^2^ in SCI, n = 3, respectively). PDGFRα^+^ cells also decreased to 53% (D3) and 35% (D7) after SCI in PDGFRα/eGFP mice, (Fig. 3A–C). We confirmed these findings by Western analysis and verified reduction in PDGFRα protein in detrusor muscles of SCI mice (n = 4, Fig. 3D,E). These findings were consistent with the transcriptional changes observed after SCI (see Fig. 1), and suggest overall reduction in PDGFRα^+^ cells and reduced expression of SK3 that would negatively impact the regulation of excitability provided by PDGFRα^+^ cells in the bladder.
3. Apoptosis of PDGFRα^+^ cells in SCI
  We examined changes in the expression of apoptosis pathways to better understand the fate of PDGFRα^+^ cells after SCI. RNA-seq of whole muscle samples showed geneset scores computed for the apoptosis-related KEGG and GOBP terms, respectively, using the FAIME algorithm^22^. The apoptosis-related geneset score was significantly increased (t-test: *P* < 0.05) in detrusor muscles after SCI (Fig. 4A). Apoptosis-related genes, including *Apaf1, Capns1, and Casp3*, were significantly upregulated (FC > 1.5 and *FDR* < 0.05) in detrusor muscles SCI D3 (Fig. 4B). Time dependent increases in expression of *Apaf1* (Fig. 4C) and *Caspase3* (Fig, 4D) in detrusor muscles after SCI (as compared to control; n = 4 for each period) were confirmed by qPCR.
4. Ex vivo preparation to confirm the role of PDGFRα^+^ cells in SCI
  Ex vivo bladder preparations were used to characterize the relationship between intravesical volumes and pressures in bladders after SCI. Ex vivo preparation can exclude extrinsic neural reflexes during filling. SK channels are highly expressed in detrusor PDGFRα+ cells, and antagonists of these channels increase TCs during filling17. We examined the effects of the SK channel antagonist, Apamin (300 nM) on bladders from control and after SCI (up to 1 Mo).

**Figure 5:**
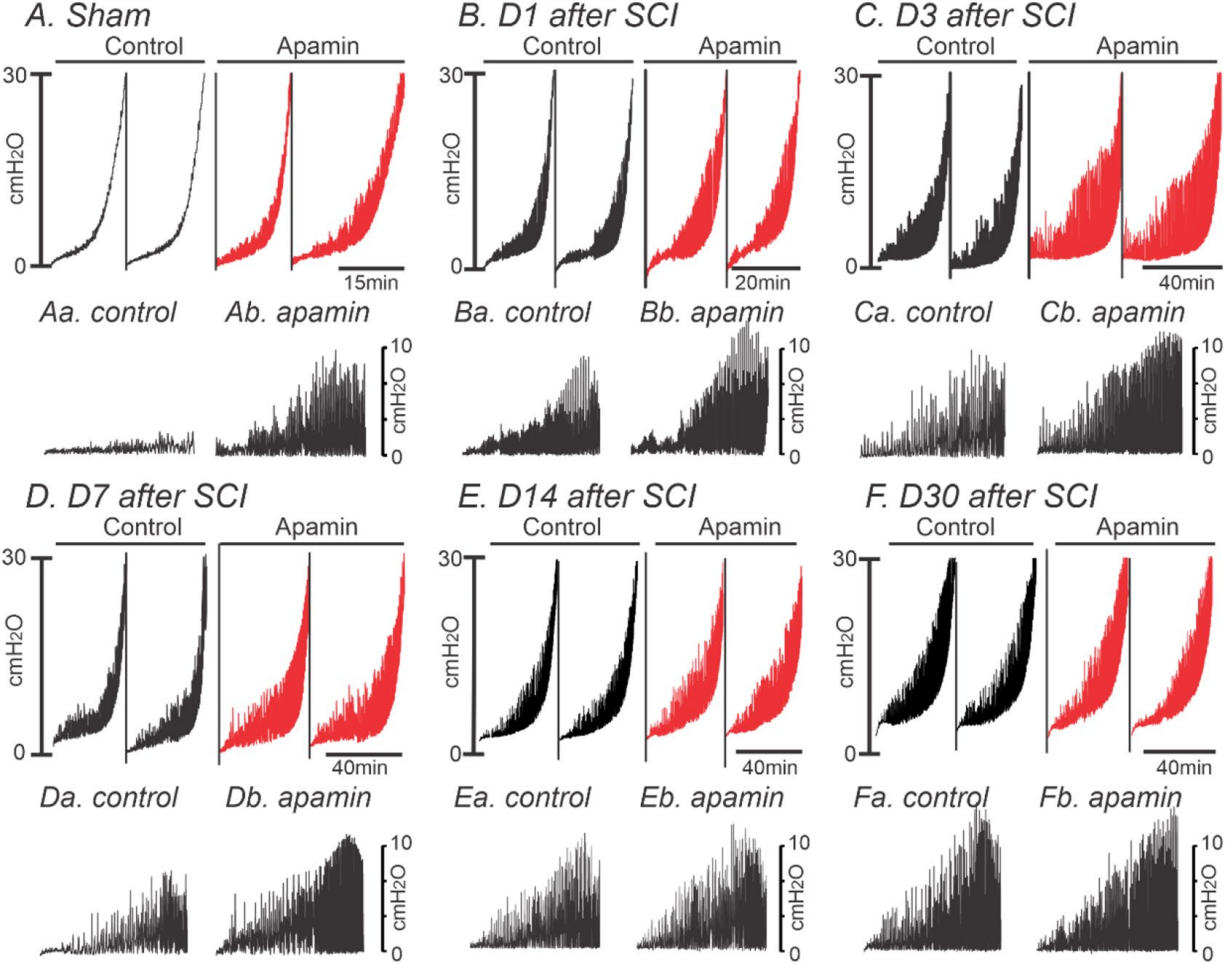
The effects of apamin on transient contractions (TCs) of sham, D1, D3, D7, D14 and D30 after SCI using ex vivo preparation. ***(A–F)*** Ex vivo pressure-response curve for control and apamin application. (**Aa,b–Fa,b**) Expanded time scales with adjustment of baseline under control (**a**) and apamin (**b**) from above panels.

In control preparations, infusion of Krebs–Ringer bicarbonate solution (20 µl/min) generated repeatable responses consisting of small amplitude, low frequency of TCs (Fig. 5A). Apamin (300 nM added to the bathing solution) increased the amplitude (6.9 ± 1.2 cmH_2_O, P < 0.01) and frequency (65 ± 12 events, P < 0.01, n = 11) of TCs during bladder filling, as compared to control (2.1 ± 0.5 cmH_2_O in amplitude and 22 ± 9.1 events in frequency; Fig. 5A, Table 1). At D1, D3, D7, D14 and D30 after SCI, the amplitude and frequency of TCs during filling were increased (Fig. 5B–F, Table 1). Enhanced TCs during filling persisted for at least 30 days after SCI, and apamin failed to induce significant changes of amplitude (from D7) and frequency (from D1) of TCs after SCI (Fig. 5B–F, Table 1) indicating that development of DO after SCI progressed for up to 1 month after SCI. Bladder capacity and filling time to reach 30 cmH_2_O were also increased in all of SCI groups compared to control (Table 2). The enlarged bladder capacity due to lack of voluntary voiding and prolonged infusion time was prominent at 30 days after SCI.

**Table 1:** Summarized data of frequency and amplitude before and after apamin.

	Frequency (events per min)		Peak amplitude (cmH_2_O)	
	Control	Apamin	Control	Apamin
Control (n = 11)	22 ± 9.1	65 ± 12.0^#^	2.1 ± 0.5	6.9 ± 1.2^#^
D1 (n = 10)	52 ± 9.7*	71 ± 13.4	4.8 ± 1.2*	7.2 ± 1.5^#^
D3(n = 12)	56 ± 11.1*	69 ± 11.7	5.1 ± 0.6*	7.8 ± 1.1^#^
D7 (n = 9)	55 ± 10.8*	64 ± 9.2	5.9 ± 1.2*	7.2 ± 1.4
D14 (n = 6)	67 ± 10.4*	69 ± 11.6	7.0 ± 1.3*	8.3 ± 2.3
D30 (n = 6)	96 ± 6.7*	98 ± 4.1	6.6 ± 0.7*	6.9 ± 0.5

*P < 0.05 denotes comparison between control and each period after SCI.

^#^P < 0.05 denotes comparison of effects of apamin at each period.

**Table 2:** Summarized data of infusion volume and time to reach 30cmH_2_0.

	Infusion volume (µl)	Infusion time (min)
Control (n = 11)	298 ± 53	11.9 ± 2.0
D1 (n = 10)	432 ± 95*	24.3 ± 4.1*
D3 (n = 12)	568 ± 105*	23.5 ± 4.7*
D7 (n = 9)	539 ± 78*	24.1 ± 3.9*
D14 (n = 6)	905 ± 126*	36.2 ± 5.0*
D30 (n = 6)	1108 ± 67*	41.2 ± 2.6*

*P < 0.05 denotes comparison between control and each period after SCI.

## Discussion

In this study we investigated responses of the murine bladder and the status of PDGFRα^+^ cells after SCI. PDGFRα^+^ cells and the regulatory functions provided by these cells in regulating bladder contractions during filling were greatly decreased after SCI. TCs increased dramatically during bladder filling, and accompanying this change in function, *Pdgfra* and *Kcnn3* expression decreased. PDGFRα and SK3 proteins in detrusor muscles also decreased. Loss of PDGFRα^+^ cells due to apoptosis was also observed in detrusor muscles. The increase in TCs was associated with decreased sensitivity to apamin and SKA-31 during bladder filling, which is consistent with the reduced expression of SK3 and loss of PDGFRα^+^ cells that express SK3 channels. These findings demonstrate a novel mechanism for development of DO after SCI that is linked to loss of the inhibitory regulation provided by PDGFRα^+^ cells during bladder filling.

PDGFRα^+^ cells regulate detrusor excitability during bladder filling^23^. SK channel antagonists potentiate the amplitude of spontaneous contractions in murine^24^, guinea pig^25^ and human^26^ detrusor muscles. The effects of apamin are likely due to blocking SK channels in PDGFRα^+^ cells which have a high expression of SK3 channels, because the current density from SK channels is minimal in SMCs and not resolvable in murine SMCs at physiological potentials^27^. Thus, PDGFRα^+^ cells express a powerful mechanism to suppress excitability and allow the bladder to fill with minimal activation of TCs and sensory discharge^23^. Downregulation of *Pdgfra* and *Kcnn3* genes occurred in detrusor PDGFRα^+^ cells and can explain the development of DO after SCI. Indeed, transcriptional analysis revealed downregulation of *Pdgfra* and *Kcnn3* in early stage of SCI bladder. In contrast the phenotype of SMCs seemed more stable after SCI, since no changes were observed in key genes, such as *Myh11, Kcma1* and *Cacna1c.*

IHC showed that the distribution of PDGFRα^+^ cells decreases in detrusor muscles after SCI. The immunohistochemistry findings were confirmed using a reporter strain of mice with expression of eGFP in nuclei of PDGFRα^+^ cells, as a relatively low density of eGFP^+^ cells was found in bladders of PDGFRα/eGFP mice after SCI. However, IHC is not reliable method to quantify loss of protein, so protein expression in extracts of detrusor muscles was measured by Western analyses. Western blots showed the downregulation of PDGFRα expression and SK3 expression which was mirrored by transcriptional expression.

Loss of PDGFRα^+^ cells caused by SCI were due to apoptotic changes in detrusor PDGFRα^+^ cells although the mechanism inducing apoptosis in detrusor PDGFRα^+^ cells by SCI has not been elucidated. *Apaf1* as well as *caspase3* increased after SCI in comparison to control indicating that cell death occurred as a result of SCI. The mechanism causing damage to the regulatory function provided by PDGFRα^+^ cells after SCI is an important topic for future research.

Ex vivo preparations were used in the present study to isolate bladders and exclude connections from central and spinal reflexes. Release of mediators from the urothelium may also influence detrusor excitability, but substances and receptors involved in such a mechanism have not been identified. Ex vivo bladder developed increased TCs after SCI as compared to control bladders (see Table 1), and enhanced TCs persisted for at least 30 days after SCI. Effects of apamin on responses to bladder filling remained for up to 72 h after SCI, but the sensitivity to apamin decreased after SCI due to partial loss of PDGFRα^+^ cells and downregulation of SK3 channels. These findings, observed in ex vivo bladders, describe a novel myogenic mechanism that is sufficient for development of DO after SCI.

SCI patients have less opportunity to see a urologist due to other complications that often require more urgent attention than bladder dysfunction. This prevents patients from early and appropriate examination and treatment of lower urinary tract dysfunction in the early phase of SCI. Although there are many reports of DO after recovery from spinal shock^28–33^, only a few reports confirm development of DO in the acute phase of SCI (i.e. 3–40 days after SCI)^34,35^. During the period of spinal shock (1-2wks after SCI in rodents), the initial areflexia bladder and urinary retention occur due to a lack of voiding contractions^36–38^. Unfortunately, the initial urodynamic changes during the spinal shock phase have not been investigated due to possible complications^39^. Thus, convincing in vivo cystometry data characterizing bladder function during the spinal shock phase after SCI are lacking. Although there are many reports of DO after recovery from spinal shock^30–35^, we do not know the incidence of DO in human patients during the spinal shock period. Increased NVCs are a sign of DO, and NVCs can be observed within 1 week in rodents after SCI^40^. The current study showed that DO develops during spinal shock and is sustained after recovery after spinal shock in the animal model used.

Early treatment to avoid higher intravesical pressure with lower bladder compliance followed by vesicoureteral reflux associated with DO has been suggested to keep patients’ renal and bladder function serving as a ‘low pressure tank’ without waiting for the irreversible complications of SCI^34,35^. Given the importance of an early intervention for a treatment especially focusing on a myogenic aspect, preventing the phenotypic change of PDGFRα^+^ cells and rescuing the function of SK channels in bladder PDGFRα^+^ cells might be a promising target to avoid development of DO after SCI.

## Methods

### Spinal cord injury (SCI) animal model

All experimental procedures were conducted in accordance with the National Institutes of Health *Guide for the Care and Use of Laboratory Animals* and the animal use protocol, reviewed and approved by the Institutional Animal Use and Care Committee at the University of Nevada. All methods are reported in accordance with ARRIVE guidelines. C57BL/6 (male mice, 8–12 wks old), *Pdgfra*^*tm11(EGFP)Sor*^/J (PDGFRα/eGFP, Jackson lab) and smMHC/Cre/eGFP (SMC/eGFP) from Jackson Lab used for SCI operations and age-matched control. Laminectomies were performed under isoflurane anesthesia (3—4% with a balance of oxygen for induction followed by 2% for maintenance), and the spinal cord (T11–T13) were exposed without any damage or compression to the surrounding dura. Dumont #5 forceps were positioned in the middle of the exposed spinal cord segment to perform complete spinal cord transection. Complete spinal cord transection was done at T 12 confirmed by retraction of rostal and caudal cut ends of spinal cord under surgical microscope, which had a space approximately at 2 mm. Control animals received sham operations with exposing the vertebrae at same level as SCI without damaging any spinal cord and dura. Enrofloxacin (5 mg/kg) was applied subcutaneously for three days after SCI followed by twice a week after SCI surgery until ex vivo or molecular evaluation was done. The bladder was manually squeezed to eliminate the residual urine of bladder once daily. Bladders were collected for experiments at D1, D3, D7, D14 and D30 after SCI surgery and in sham control.

### Ex vivo preparation

Bladders were removed. A PE50 catheter (Intramedic, Fisher Scientific, Santa Clara, CA) with a cuff was placed in the urethral opening and ligated tightly with silk thread just above the ureterovesical junction and constant monitoring of pressure. Intravesical pressure were recorded with reference to atmospheric pressure (p = 0) at the level of the bladder connected to a quad-bridge amplifier (AD Instruments) interfaced to a computer. Krebs–Ringer bicarbonate (KRB) solution (37 °C) was infused (25 µl/min) and stopped when bladder pressures reach 30–40 cm/H_2_O to avoid a permanent damage^4^. At least 3 fills will be performed under each experimental condition to ensure reproducibility. The effect of apamin was tested on control and spinal cord injured bladder. Ex vivo data were captured using the threshold search by Clampfit 10 (Molecular Device) with baseline adjustment to examine the frequency and amplitude of transient contractions occurring during the filling phase.

### Molecular preparation

Dissection of detrusor smooth muscles and RNA isolation in control and SCI were identical as previously described^18^. For quantitative analysis of transcripts, PDGFRα^+^ cells and eGFP/SMCs were purified by fluorescence‐activated cell sorting and detrusor muscles for molecular tests. Total RNA was isolated from detrusor muscles, PDGFRα^+^ cells and eGFP/SMCs using illustra RNAspin Mini RNA Isolation kit (GE Healthcare, Little Chalfont, UK), and first‐strand cDNA was synthesized using SuperScript III (Invitrogen, Carlsbad, CA, USA), according to the manufacturer's instructions. PCR was performed with specific primers using Go‐Taq Green Master Mix (Promega Corp., Madison, WI, USA). The following PCR primers designed against murine sequences were used (GenBank accession number is given in parentheses for the reference nucleotide sequence used): *Pdgfra* (NM_011058) and *Kcnn3* (NM_080466). Quantitative PCR (qPCR) was performed with the same primers as PCR using Fast SYBR Green chemistry (Applied Biosystems, Foster City, CA, USA) on the 7900HT Real Time PCR System (Applied Biosystems). Regression analysis of the mean values of three multiplex qPCRs for the log_10_‐diluted cDNA was used to generate standard curves. Unknown amounts of messenger RNA (mRNA) were plotted relative to the standard curve for each set of primers and graphically plotted using Microsoft Excel. This gave transcriptional quantification of each gene relative to the endogenous glyceraldehyde 3‐phosphate dehydrogenase (*Gapdh*) standard after log transformation of the corresponding raw data.

Transcriptomes profiled by mRNA-seq (Novogene Co Ltd) were investigated to identify the genes and pathways potentially involved in the regulation of excitability in PDGFRα^+^ cells upon SCI treatment. Total RNA was obtained from detrusor muscles in control and SCI. Using the SAM tool^41^, the genes with false discovery rate (FDR) < 5% and fold change (FC) > 1.5% were deemed to be differentially expressed. The *FAIME* algorithm^22^ was applied to assign gene expression-based geneset scores for the “apoptosis” related genes defined by both the Kyoto Encyclopedia of Genes and Genomes (KEGG)^42^ and Gene Ontology (GO)^43^ databases. The *FAIME* method generates geneset scores using the rank-weighted gene expression of individual samples, which determines whether an a priori defined set of genes shows statistically significant, concordant expression differences between two biological states (e.g. control vs. SCI), and provides a mechanistic interpretation of the deregulated genes.

### Whole mount immunohistochemistry

C57BL/6 and *Pdgfra*^*tm11(EGFP)Sor*^/J bladders were cut open from the neck up to the dome. Tissues were dissected in Krebs ringer solution containing 118.5 mM NaCl, 4.7 mM KCl, 2.5 mM CaCl_2_, 1.2 mM MgCl_2_, 23.8 mM NaHCO_3_, 1.2 mM KH_2_PO4 and 11 mM dextrose, then pinned down on Sylgard dish and stretched 150% from the resting state. For urothelial denudation, urothelium was removed and surface of the muscle was scraped to remove any residual sub‐urothelial cells. Fixation and incubation of tissues were identical as previously described^16^. For double labelling studies, tissues were re‐blocked for 1 h in 10% normal donkey serum (Sigma‐Aldrich) and incubated overnight in antibody of choice, diluted in 0.5% Triton‐X (Sigma‐Aldrich) and incubated in the appropriate Alexa Fluor (Invitrogen) antibody diluted 1:1000 in PBS for 1 h. Processed tissues were mounted with Aqua mount mounting media (Lerner Laboratories, Pittsburgh, PA, USA) on glass slides and cover slipped and imaged. The primary antibodies of PDGFRα (R&D Systems, Inc.) and SK3 (Alamone Labs) were used and primary antibodies were omitted in the procedure for negative controls.

### Automated capillary electrophoresis and chemiluminescent western blotting

Muscles were snap-frozen in liquid N_2_, and stored at − 80 °C. For analysis, the methods for homogenization, centrifugation and collection of the supernatants were identical as previously reported^44,45^. Protein concentrations were determined by Bradford assay ^45^. Analysis of protein expression was performed according to the User Guide using a ProteinSimple Wes instrument (CA, USA). Each sample was mixed with fluorescent 5 × Master Mix, incubated at 95 °C for 5 min and then loaded into a Wes 12–230 kDa prefilled plate, along with a biotinylated protein ladder, blocking buffer, primary antibodies, ProteinSimple HRP-conjugated anti-rabbit secondary antibody, luminol peroxide, and washing buffer. The plates and capillary cartridges were placed into the Wes for electrophoresis and chemiluminescence immunodetection by a CCD camera using default settings. Compass software was used to acquire and analyze the data and generate gel images and chemiluminescence intensities. Protein expression levels are expressed as the chemiluminescence intensity area under the primary antibody peak per µg protein.

### Drugs

All reagents were purchased from Sigma‐Aldrich (St Louis, MO, USA) and apamin (Tocris, UK) solubilized in the bath solution for ex vivo recordings.

### Statistical analyses

All data are expressed as means ± SEM. “n” represents the number of experiments. All statistical analyses were performed using Graphpad Prism. A paired and unpaired Student's *t* test was used to compare groups of data and differences were considered to be significant at *P* < 0.05.
